# A Rare Case of Severe Congenital RYR1-Associated Myopathy

**DOI:** 10.1155/2018/6184185

**Published:** 2018-08-01

**Authors:** Nicola Laforgia, Manuela Capozza, Lucrezia De Cosmo, Antonio Di Mauro, Maria Elisabetta Baldassarre, Francesca Mercadante, Anna Laura Torella, Vincenzo Nigro, Nicoletta Resta

**Affiliations:** ^1^Neonatology and Neonatal Intensive Care Unit, Department of Biomedical Science and Human Oncology, “Aldo Moro” University of Bari, Policlinico Hospital, Piazza Giulio Cesare n. 11, 70124 Bari, Italy; ^2^Medical Genetics Unit, Department of Biomedical Sciences and Human Oncology, “Aldo Moro” University of Bari, Policlinico Hospital, Piazza Giulio Cesare n. 11, 70124 Bari, Italy; ^3^Medical Genetics Laboratory, Department of Precision Medicine, “L.Vanvitelli” University of Campania, Telethon Institute of Genetics and Medicine (TIGEM), Naples, Italy

## Abstract

Congenital myopathies are a group of rare inherited diseases, defined by hypotonia and muscle weakness. We report clinical and genetic characteristics of a male preterm newborn, whose phenotype was characterized by severe hypotonia and hyporeactivity, serious respiratory distress syndrome that required mechanical ventilation, clubfoot, and other dysmorphic features. The diagnostic procedure was completed with the complete exome sequencing of the proband and of his parents and his sister, which showed new mutations in the ryanodine receptor gene (RYR1), which maps to chromosome 19q13.2 and encodes the skeletal muscle isoform of a calcium-release channel in the sarcoplasmic reticulum (RyR1). This report confirms that early diagnosis and accurate study of genomic disorders are very important, enabling proper genetic counselling of the reproductive risk, as well as disease prognosis and patient management.

## 1. Introduction

Congenital myopathies are a group of rare inherited diseases, defined by hypotonia and muscle weakness, that usually present at birth or early childhood, in association with characteristic morphological defects. They are caused by genetic mutations of the structural proteins of skeletal muscle and present variable inheritance: dominant, recessive, or X-linked [1, 2].

Mutations in a single gene may be associated with different clinical and histological findings, and the same clinical or histological conditions may be due to mutations in different genes [3]. Phenotypic findings include severe neonatal-onset, mild forms with nonprogressive weakness, and muscle hypotonia with later onset.

The old classification of congenital myopathies was mainly based on histological features observed in the muscle biopsy [1, 3]: myopathies with “rods” (nemaline myopathies), myopathies with centralized nuclei (myotubular and centronuclear myopathies), myopathies with fiber type disproportion, and myopathies with “cores” (oval areas in the muscle cells) [4, 5]. The last ones are the most common forms of congenital myopathies, in particular the “central core” myopathy (CCD). The most known CCD is the autosomal dominant inherited form caused by mutations localized in three hot spots in the ryanodine receptor 1 gene (RYR1), associated with malignant hyperthermia susceptibility (MHS) and clinically characterized by minor hypotonia and nonprogressive weakness. Recessive mutations are located throughout the entire RYR1 gene and characterized by a more severe and progressive clinical presentation with neonatal-onset and significant generalized muscle weakness [6–8]_._

Commoner myopathy disorders presenting in fetal or neonatal period are shown in Table 1.

## 2. Ryanodine Receptor 1 Gene (RYR1)

RYRs are a family of intracellular calcium (Ca^2+^) release channels that allow rapid release of Ca^2+^ from sarcoplasmic reticulum (SR) into the cytosol, crucial for heart and skeletal muscle contraction. Three mammalian isoforms (RYR1, RYR2, and RYR3) exhibit subtype-specific tissue expression patterns. RYR1 is predominant in skeletal muscle while RYR2 is exclusively expressed in cardiac myocytes. RYR3 is involved in skeletal muscle development but the exact role is still unclear and studies based on murine knockout propose an involvement in learning and memory [9].

The ryanodine receptor 1 gene maps to chromosome 19q13.2 and encodes the skeletal muscle isoform of a calcium-release channel in the sarcoplasmic reticulum (RyR1) [6, 10]. It has been involved in both dominant and recessive congenital myopathies and it plays a central role in excitation-contraction coupling, causing altered excitability and/or changes in calcium homeostasis in muscle cells [11–14].

RYR1 and RYR2 function is controlled by Cav1.1, also known as dihydropyridine receptor (DHPR). During the potential action, the Cav1.1-coupled RYR1 channels release the SR Ca^2+^ required for muscle contraction. During depolarization, RYR1 is inactivated by a Ca^2+^ mediated mechanism and Cav1.1 returns to a closed state. Conformational changes in the DHPR induce RYR1 opening. Ca^2+^ release is subsequently decreased during depolarization by Ca^2+^-induced inactivation of RYR1, a negative feedback mechanism, and eventually terminated by membrane repolarization, which drives the return of Cav1.1 to a closed and resting state by reversing the activation of the Cav1.1 voltage sensor [15, 16].

Recent advances suggest that abnormal excitation-contraction coupling may be a common theme in the congenital myopathies [17].

The central role of RYR1 in regulating Ca^2+^ release elucidates the relationship between muscle pathologies and mutations in this gene. The main channelopathies due to mutation in RYR1 are susceptibility to malignant hyperthermia (MSH) and central core disease (CCD). Mutations in RYR1 are also associated with other myopathies as multiminicore disease (MmD), nemaline myopathy, and centronuclear myopathy.

The most mutations in RYR1 linked to MH and/or dominant CCD are missense substitutions and are conserved in three “hot spots” located in the N-terminal (aminoacid residues 2,163–2,458; exons 1-17), central (amino acid 35–614 2,163–2,458; exons 39-46), and C-terminal regions (aminoacid residues 4,550–4,940; exons 90-104) in the aminoacid sequence of RyR1 [8, 18]. However, many mutations are located outside these hot spots. MSH is usually associated with mutations in N-terminal and central regions and it is inherited in a dominant way. CCD can be transmitted in dominant or recessive manner. Dominant mutations are frequently located in the hot spots, especially in C-terminal region, while recessive mutations are distributed throughout the entire coding sequence and are correlated to earlier and more severe presentation. Recessive mutations can be missense, which result in the production of a functionally deficient RyR1 protein (loss of function) or hypomorphic mutations (nonsense, frameshift, and splice), which cause reduction of RYR1 expression (lowering mRNA levels) with marked reduced or absent protein expression. In recent studies, evidences of recessive mutations are increased by the reason that until recently RYR1 was not screened entirely, but the analysis was limited to the hot spots regions [7, 18].

## 3. Clinical Report

The proband is a male newborn, the third son of Caucasian nonconsanguineous parents, born preterm at 34 weeks via caesarean section for polyhydramnios. He had an intrauterine growth restriction, with birth weight of 1690 g (<10th p) and length of 42 cm (<10th p); head circumference was 33 cm (=85°p). The newborn had two brothers who died in the neonatal period: a male (33 weeks), with bilateral clubfoot and exitus in the fourth day of life for respiratory failure, a female (33 weeks), without malformations but with exitus after 4 hours of birth. Both pregnancies were complicated by polyhydramnios, responsible for preterm caesarean section.

Severe hypotonia was noted after birth and perinatal period was remarkable for serious respiratory distress syndrome that required mechanical ventilation. Physical examination showed clubfoot, low-set ears with dysplastic lobes and dysmorphic antihelix, anteverted nostrils, arachnodactyly, low-set thumbs, bilateral clinodactyly of 5th finger of the hands, and varus foot. No anomaly was evident in thoracic auscultation and abdominal palpation. The neurological objectivity was characterized by persistent global hypotonia (“floppy baby”), generalized muscle weakness, hyporeactivity, absence of sucking reflex, tendon reflexes, and spontaneous motility, and minimal response of the distal and of the lower limbs after painful stimulation.

Complete blood count, C-reactive protein, serum electrolytes, renal function, lactic acid levels, ammonia levels, aminoacidemia, and PCR-research for CMV in urine were normal. Slight increase in AST (194 U/l), ALT (210 U/l), *ɣ*GT (363 U/l), and CPK (356 U/L). Investigations including cranial ultrasound, fundus examination, cardiac and renal ultrasound, karyotype, and genetic testing for SMA-1 and SMARD were performed and they were normal.

Chest X-ray showed a displaced fracture of the middle third of the proximal femoral shaft with stumps straddle and a compound fracture of the middle third of the diaphysis of the left humerus and thin ribs.

EEG showed a discontinuous pattern of medium voltage, immature for chronological age of the patient.

EMG performed at 9 days of age at the level of the left tibial muscle revealed a myopathic pattern. Muscle biopsy, performed at the first day of life, in the femoral quadriceps, showed total absence of muscle tissue. Biopsy of deltoid muscle was not possible, because there was no muscle tissue at inspection. A second biopsy was needed, performed at 12 days of age in the triceps, which showed great histologic heterogeneity, including marked variability of the size of muscle fibers, atrophic fibers, fibrosis, adipose tissue infiltration, and increased connective tissue without cores, which may not be present in recessive RYR1 mutations.

Both enzyme histochemistry and electron microscopy were performed and they did not reveal cores.

Depending on the age of the patient, the proportion of nuclear centralization was normal. Immunohistochemical analysis did not reveal significant alterations of the muscle proteins.

It is important to remember that clinical severity may not correlate with the degree of histologic change.

The diagnostic procedure was completed with the complete exome sequencing of the proband and of his parents and his dead sister, thanks to the Guthrie Card.

Next-generation sequencing of our proband's complete exome revealed two variants in the RYR1 gene: NC 000019.9:g.38964345del (p.Gly1365Glufs*∗*33) and NC 000019.9:g.39076790C>G (p.Phe4976Leu), sited, respectively, in exons 28 and 104. Sanger sequencing confirmed the results.

Segregation analysis performed by Sanger sequencing of exons 28 and 104 in the parents showed that they were disposed in two different alleles in trans: the mother inherited first mutation, while the second one had paternal origin. Thanks to the Guthrie Card of the birth screening, it was possible to extract dead sister's DNA and perform Sanger sequencing of exons 28 and 104, which showed the same result found in the proband. No biological samples were available for the first son.

Our proband is a compound heterozygous for the c.4094del G hypomorphic mutation and the c.14928C>G missense mutation.

The hypomorphic mutation, a frameshift deletion leading to a stop codon in the reading sequence (NC 000019.9:g.38964345del), is located in exon 28, thereabout the N-terminal region (but not in the N-terminal hot spot), and it has never been reported before, to our knowledge.

The missense mutation (NC 000019.9:g.39076790C>G) falls in exon 104 in the C-terminal region.

During the stay in NICU, his clinical conditions have been increasingly critical, with worsening respiratory failure and permanent ventilatory assistance until death, at one month of age.

## 4. Discussion

Core myopathies are characterized by regions in muscle fibers lacking histochemical oxidative and glycolytic enzymatic activity, reflecting absence of mitochondria that appear as “cores” on oxidative stains.

In RYR1 related dominant CCD, cores are large and with a longitudinal extension. In MmD, associated with recessive mutations, cores are shorter and both type I and type II muscle fibers are involved without a myofibrillar organization. The specific underlying process that causes the core formation is still unknown [15, 17, 19].

Dominant mutations have traditionally been associated with central CCD and/or susceptibility to malignant hyperthermia [8], while much less is known about recessive mutations and their mechanism of disease [20]. Core myopathy is probably the most common congenital myopathy but may still be underrecognized because the characteristic histopathological changes may not be present on biopsies at an early age because cores or other core-like areas may develop over time, with an age-related appearance [17, 19–21].

Recently Snoeck and colleagues analysed a cohort of 77 nonrelated patients affected by RYR1 related myopathies focusing on the evolution of phenotype throughout life. Even if some changes can be detectable from the first histopathological analysis, serial biopsies performed in the same patients revealed that other diagnostic and typical features, especially the presence of cores, occurred in later biopsies [22].

More than 300 mutations in RYR 1 have been associated with various forms of neuromuscular disorders with high variability in clinical and histological features, showing complex genotype-phenotype correlations associated with RYR 1 mutations [23–25]. Both dominant and recessive mutations of the RYR1 gene can result in a severe neonatal-onset phenotype. More clinical and histologic heterogeneity has been seen in patients with recessive RYR1 gene mutations, in which central cores are not obligatory histologic features. The absence of cores should not preclude consideration of a RYR1-associated myopathy, as age at biopsy and site of biopsy may influence the detection of classic cores.

The molecular mechanisms by which dominant versus recessive mutations lead to ryanodine receptor dysfunction and myopathy are not completely clear. Dominant mutations associated with CCD are clustered in the C-terminal portion of RYR1 and likely result in functional abnormalities of the intracellular calcium-release channel. In contrast, a clear clustering of recessive RYR1 mutations is not yet clearly apparent [21].

In 2012 Klein and colleagues have described a severe neonatal form associated with recessive mutations in the RYR 1 gene, characterized by severe clinical presentation, early onset, and significant generalized muscle weakness [7]. As previously reported, recessive mutations, associated with variable histological patterns and symptoms, can be located throughout all gene [14]. Recessive RYR 1 mutations can be both missense mutations compared to hypomorphic mutations. Missense mutations result in the production of a functionally deficient RyR1 protein. Hypomorphic mutations (nonsense, frameshift, and splice) cause reduction in the levels of mRNA and in protein expression, even if some residual RYR 1 function is indispensable for life [22, 26, 27]. Furthermore, some studies showed a statistically significant association between the presence of a hypomorphic mutation and a severe clinical picture. In congenital myopathies reduced total RYR 1 protein levels are an important disease mechanism that heralds more severe disease.

In 2013, Amburgey and colleagues studied genotype-phenotype correlations in recessive RYR-related myopathies, correlating type and location of the mutation to clinical and histopathologic presentation [24]. The presence of a hypomorphic allele, which can be found in any part of RYR1 gene, was clearly correlated to increased clinical severity and ophthalmoparesis. Conversely, nonhypomorphic mutations, particularly missense mutations, are more often located in the MH/CCD hotspots, particularly in the C-terminal one (hotspot 3). The analysis of nonhypomorphic mutations showed that they are usually correlated to a milder phenotype, except for those falling in hotspot 3, which are associated with a more severe phenotype, even at the heterozygous state [18].

While the hypomorphic mutation of the proband has never been reported before, the missense mutation is reported in 1000 Genome Browser as extremely rare in the general population and in ClinVar database (rs 368874586) as having pathogenic/likely pathogenic significance. In their study about exome sequencing of 500 family with undiagnosed conditions, Farrel and collaborators reported this mutation associated with an unexpected inborn genetic disease with autosomal recessive inheritance [28].

## 5. Conclusions

A high index of suspicion and appropriate genetic testing can help in diagnosis of specific type of congenital myopathy. An appropriate diagnosis results in better genetic counselling and also opens possibilities for prenatal diagnosis.

## Figures and Tables

**Table 1:** Commoner myopathy disorders presenting in fetal and/or neonatal period.

**Congenital myopathies**	**Histological features**	**Clinical ** **features**	**Genes Mutated**	**Inheritance**
**MYOPATHIES WITH “RODS” (NEMALINE MYOPATHIES)**	Presence of nemaline bodies or rods (protein aggregates)	Diaphragm weakness, distal weakness in lower extremities, congenital arthrogryposis, severe facial and bulbar weakness, severe hypotonia, and hyporeactivity, progressive respiratory failure	TPM3 (1q21.3) NEB (2q22.3) ACTA 1 (1q42.13) TPM2 (9p13.3) TNNT1 (19q13.42) KBTBD 13 (15q22.31) CFL2 (14q13.1)	AD or AR AR AD or sporadic, AR AD AR(Amish private mutation) AD AR

**MYOPATHIES WITH CENTRALIZED NUCLEI **				
**Myotubular myopathies (XLMTM)**	Muscle fibers similar to myotubes (normally observed at 8-15 weeks' gestation)	Severe prenatal or neonatal myopathy, reduced fetal movements, polyhydramnios, swallowing difficulties, severe generalized hypotonia and weakness from birth, progressive ophthalmoparesis, severe respiratory insufficiency requiring ventilator support	MTM1 (Xq28)	Recessive X-linked
**Centronuclear myopathies**	High incidence of centrally and/or internally placed nuclei in rows in muscle fibers	Progressive ophthalmoparesis, early respiratory failure, progressive craniofacial deformities	DNM2 (19q13.1) MYF6 (12q21.31) CCDC78 (16p13.3) BIN1 (2q14.3)	AD AD AD AR

**MYOPATHIES WITH FIBER TYPE DISPROPORTION**	Type 1 fibers are consistently and significantly smaller (hypotrophic) than type 2 fibers. Disproporzione del calibro delle fibre: fibre del tipo 1 (fibre lente) sono più piccole (almeno del 40%) rispetto alle fibre del tipo 2 (fibre veloci)	Low tone without other distinguishing characteristics	ACTA1 (1q42.1) Locus 2 (Xq13.1-q22.1) SEPN1 (1p36.11) TPM3 (1q21.2) TPM2 (9p13) MYL2 (12q24.11)	AD AR AR AD AD AR

**MYOPATHIES WITH “CORES” **	C*ores*: well-delimited, rounded areas devoid of oxidative staining, located in the cytoplasm of the muscle fibers.			
**Central core myopathy**	Single and central cores in muscle fibers	Decreased fetal movement during pregnancy, muscle pain/cramps, global hypotonia, weakness of the facial muscles, severe skeletal malformations, possible malignant hyperthermia	RYR1 (19q13.1) SEPN1 (1p36.11) TTN (2q31.2) MYH7 (14q12)	AR AR or sporadic AR AD
**Multiminicore myopathy**	Multiple, short areas of sarcomere disorganization containing reduced numbers of mitochondria in skeletal muscle fibers	Neonatal hypotonia, global muscle weakness, delayed motor development	RYR1 (19q13.1) SEPN1 (1p36.11) TTN (2q31.2) MYH7 (14q12)	AR AR or sporadic AR AD
